# Comparing extraction method efficiency for high-throughput palaeoproteomic bone species identification

**DOI:** 10.1038/s41598-023-44885-y

**Published:** 2023-10-26

**Authors:** Dorothea Mylopotamitaki, Florian S. Harking, Alberto J. Taurozzi, Zandra Fagernäs, Ricardo M. Godinho, Geoff M. Smith, Marcel Weiss, Tim Schüler, Shannon P. McPherron, Harald Meller, João Cascalheira, Nuno Bicho, Jesper V. Olsen, Jean-Jacques Hublin, Frido Welker

**Affiliations:** 1 Chaire de Paléoanthropologie, CIRB (UMR 7241–U1050), Collège de France, Paris, France; 2 Max Planck Institute for Evolutionary Anthropology, Leipzig, Germany; 3 Center for Protein Research, University of Copenhagen, Copenhagen, Denmark; 4 Globe Institute, University of Copenhagen, Copenhagen, Denmark; 5 Interdisciplinary Center for Archaeology and Evolution of Human Behaviour, University of Algarve, Faro, Portugal; 6 School of Anthropology and Conservation, University of Kent, Kent, UK; 7 Institut für Ur- und Frühgeschichte, Friedrich-Alexander-Universität, Erlangen, Germany; 8 Thuringian State Office for the Preservation of Historical Monuments and Archaeology, Weimar, Germany; 9 Department of Human Origins, Max Planck Institute for Evolutionary Anthropology, Leipzig, Germany; 10 State Office for Heritage Management and Archaeology, Saxony-Anhalt—State Museum of Prehistory, Halle (Saale), Germany

**Keywords:** Proteomics, Archaeology

## Abstract

High-throughput proteomic analysis of archaeological skeletal remains provides information about past fauna community compositions and species dispersals in time and space. Archaeological skeletal remains are a finite resource, however, and therefore it becomes relevant to optimize methods of skeletal proteome extraction. Ancient proteins in bone specimens can be highly degraded and consequently, extraction methods for well-preserved or modern bone might be unsuitable for the processing of highly degraded skeletal proteomes. In this study, we compared six proteomic extraction methods on Late Pleistocene remains with variable levels of proteome preservation. We tested the accuracy of species identification, protein sequence coverage, deamidation, and the number of post-translational modifications per method. We find striking differences in obtained proteome complexity and sequence coverage, highlighting that simple acid-insoluble proteome extraction methods perform better in highly degraded contexts. For well-preserved specimens, the approach using EDTA demineralization and protease-mix proteolysis yielded a higher number of identified peptides. The protocols presented here allowed protein extraction from ancient bone with a minimum number of working steps and equipment and yielded protein extracts within three working days. We expect further development along this route to benefit large-scale screening applications of relevance to archaeological and human evolution research.

## Introduction

The majority of the archaeological skeletal record is dominated by bone specimens that cannot be assigned a species identification based solely on morphological characteristics. As a result, the increasing application of biomolecular methods over the past two decades in archaeology, palaeoanthropology, and paleontology has seen the development of genetic^1,2^ and proteomic^3–5^ methods for high-throughput taxonomic identification approaches of such bone assemblages. Proteomically, these include the development of MALDI-TOF MS-based peptide mass fingerprinting (PMF) of collagen type I^4^, data-independent acquisition (DIA)-based species identification based on limited bone proteome sequence databases^3^, or the in-depth characterization of entire skeletal proteomes^5–7^.

In particular, the application of collagen PMF (also known as Zooarchaeology by Mass Spectrometry, or ZooMS) to skeletal remains has found widespread adoption in the proteomic screening of skeletal assemblages in archaeological contexts. Studies have demonstrated that large-scale screening of thousands of bone fragments is feasible^8,9^ in different geographic regions across the globe. Such studies have provided information on the ecological context in which hominins, including humans, operated^10–12^, provided new insights into hunting strategies, animal resource processing, or herd management strategies^13–16^, and the selection of particular animal taxa for the production of bone tools^17–21^. Despite its widespread adoption in the archaeological community, collagen-based PMF suffers from comparatively low taxonomic resolution and an absence of a widely adopted, computational approach to spectral identification, preventing further adoption of this approach in the wider research community.

Liquid-chromatography tandem mass spectrometry (LC–MS/MS) overcomes or minimizes the challenges associated with MALDI-TOF MS. Some of the main challenges in MALDI-TOF spectra are the manual assignment of peptide peaks for species identification that is time-consuming, the low mass resolution, and the absence of directly obtained amino acid sequence information. Together this results in taxonomic identifications that are less precise as what would, at least theoretically, be possible using LC–MS/MS analysis of the same proteomes^3^. LC–MS/MS allows the generation of MS2 spectra for which a peptide sequence is determined using a computational search program, e.g. MaxQuant^22^. From this information, the presence of specific proteins, or protein groups, are inferred. In addition, the peptide sequences can form the basis of taxonomic or phylogenetic analysis. The improvement in accuracy, speed, and sensitivity of the last generation of MS instrumentation^23^, together with the continuous growth of protein databases^24^, allows the identification of thousands of unique proteins for each MS run from low input amounts^25^. LC–MS/MS-based approaches are therefore suited to study the highly-degraded, low-quantity proteomes preserved in archaeological remains. LC–MS/MS-based studies of archaeological and anthropological materials initially focused on collagen type I, the dominant bone protein, but increasingly include entire ancient proteomes^26–30^. Despite the relatively slow rate of protein single amino acid polymorphism (SAP) accumulation, compared to nucleotide variation accumulated at the genetic level, LC–MS/MS analysis has allowed the phylogenetic analysis of ancient protein datasets for a range of animal taxa^26,29,31–35^.

Due to the experimental simplicity of mass spectrometry-based proteomics, and the capacity to process large cohorts of samples, label-free quantification approaches are most frequently used. Current MS/MS approaches largely rely on data-dependent acquisition (DDA) for precursor ion selection, according to their abundances. This maximizes the success of peptide sequence determination but limits reproducibility and quantitative potential. To resolve these issues, in recent years, several data-independent acquisition (DIA) mass spectrometric strategies, including SWATH-MS^36^ (sequential window acquisition of all theoretical fragment ion spectra), HDMSE^37^ (high definition MSE), and AIF^38^ (all-ion fragmentation), were established. DIA implements a parallel fragmentation of all precursor ions, regardless of their intensity or other characteristics, thereby enabling the establishment of a complete record of a sample^36^. DIA approaches are now in development for ancient protein analysis and offers the potential to extend the dynamic range of MS/MS data acquisition by generating data from more peptides, especially lower abundance peptides, while also improving reproducibility and quantification.

Resulting from these developments, the “Species by Proteome INvestigation” (SPIN) is a recently proposed proteomics workflow leveraging automatic approaches to LC–MS/MS data analysis in association with shorter liquid chromatography separation and DIA or DDA spectral acquisition. SPIN was proposed with a single-step protein extraction method from mineralized tissues followed by digestion using protein aggregation capture (PAC)^39,40^. Shortening the LC–MS/MS analysis to less than 10 min became possible with new LC technology^41^ and fast-scanning data-dependent or multiplexed data-independent tandem MS acquisition methods^23^. SPIN employs an automated approach to achieve a taxonomic assignment, based on protein sequence databases with gene-wise alignments. Although demonstrated to be successful in Late Pleistocene (LP) and Holocene archaeological settings, initial results show that SPIN has a comparatively low success rate for some archaeological sites^3^.

**Figure 1:**
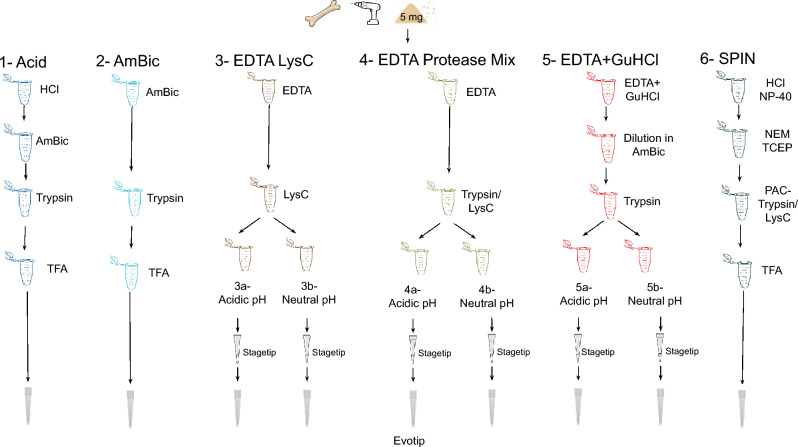
Schematic overview of extraction conditions of the six proteomic extraction methods compared in this study. The extraction approaches 3a and 5a generated no MS spectra and were excluded from further analysis.

To further explore the high-throughput capacities of SPIN proteomic analyses, we designed a comparison of six protein extraction approaches (Fig. 1). The selected extraction methods are commonly used for proteomic extractions from archaeological bone specimens^4,5,42–46^ and allow an easy scale-up for the processing of hundreds of specimens simultaneously. We apply these extraction approaches to 12 bone specimens from two Late Pleistocene (LP) cave sites with different preservation: the site Ilsenhöhle Ranis (50°39.7563’N, 11°33.9139’E, Germany, hereafter: Ranis), and Gruta da Companheira (N 37°09.19’N, 8°31.47’W, Portugal, hereafter: GdC or Companheira). Skeletal remains from LP sites are usually highly degraded and fragmented which prevents morphological species identification. Specimens from Companheira were selected as previous SPIN research at GdC indicated variable and challenging proteome preservation at the site^3^. In contrast, ongoing research at Ranis indicates excellent molecular preservation (unpublished data). This allowed us to assess the performance of the different proteome extraction methods in terms of proteome complexity, protein sequence coverage, and accuracy of species identification for two LP sites with different extents of proteomic preservation.

## Results

We analyzed 12 different bone specimens using six extraction methods in total (Fig. 1) and injected 10% of the resulting peptide digestion for LC–MS/MS analysis using an EvoSep One instrument coupled to an Exploris 480 orbitrap mass spectrometer. Six of the bone specimens are well-preserved bone specimens deriving from Ranis, Germany, and the remaining six specimens are degraded bone specimens deriving from GdC, Portugal (Table 1 and Supplementary Fig S1, SI). For extraction methods 3a and 5a, we were only able to analyze 4 specimens, including 2 blanks, due to column clogging either due to EDTA precipitation in acidic conditions or overloaded Evotips. Additionally, method 3b generated no reliable results for Ranis specimens. Consequently, methods 3a, 5a and 3b-Ranis were excluded from this study.

**Table 1:** Bone specimens used in this study.

Specimen number	Site	Layer	Chronological age	Taxonomic identity
GdC-1	Gruta da Companheira, Portugal	Galeria 1	> 50 kya	Ursidae
GdC-6	Gruta da Companheira, Portugal	Galeris 2- Level 2	> 50 kya	Ursidae/ Capra/ Bovidae/ Reindeer
GdC-7	Gruta da Companheira, Portugal	Galeria 2	> 50 kya	Not previously identified
GdC-9	Gruta da Companheira, Portugal	Galeria 2	> 50 kya	Reindeer
GdC-26	Gruta da Companheira, Portugal	Galeria 2	> 50 kya	Bovidae
GdC-193	Gruta da Companheira, Portugal	Galeria 2	> 50 kya	Not previously identified
R10300	Ilsenhöhle Ranis, Germany	X (Graue Schicht)	40–50 kya	Rhinocerotidae
R10329	Ilsenhöhle Ranis, Germany	X (Graue Schicht)	40–50 kya	Reindeer
R10340	Ilsenhöhle Ranis, Germany	X (Graue Schicht)	40–50 kya	Ursidae
R10357	Ilsenhöhle Ranis, Germany	X (Graue Schicht)	40–50 kya	Equidae
R10359	Ilsenhöhle Ranis, Germany	X (Graue Schicht)	40–50 kya	Hyaenidae/Felinae
R10363	Ilsenhöhle Ranis, Germany	X (Graue Schicht)	40–50 kya	Reindeer

The chronological age indicated is approximate and based on estimates in the archaeological literature. When available, taxonomic identity is based on prior analysis using ZooMS (for Ranis) or SPIN (for GdC).

All generated data were analysed in MaxQuant^47^ (v. 2.1.1.0) in both “specific” mode, where trypsin-specific cleavage is required at both peptide termini, and semi-specific” mode, where non-specific cleavage is allowed at one peptide terminus (see ‘Methods- MaxQuant search’). As no MS spectra were generated for both extraction methods 3a and 5a, they were excluded from the study. In addition, for the Ranis specimens, we did not generate reliable data with extraction approach 3b. We find that the “specific” search allowed the identification of approximately 250 additional MS2 spectra for Ranis compared to a “semi-specific” search, but not for GdC, while all other analyses provided identical insights (Supplementary Fig. S2, SI). Therefore, we present data on the “specific” searches below, with the comparative results of the “semi-specific” search provided in the Supplementary Information.

### Spectral acquisition

**Figure 2:**
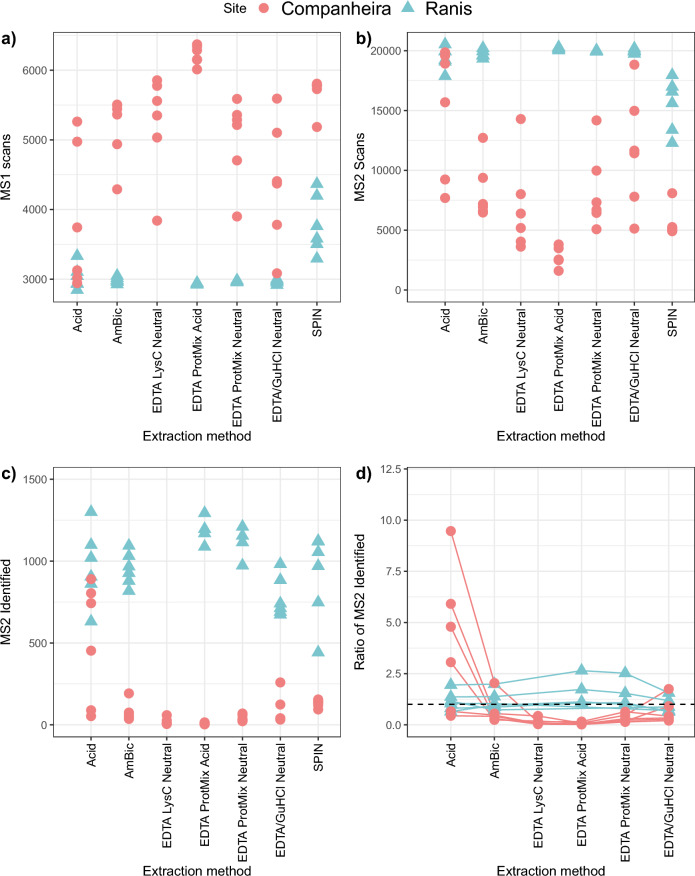
Summary information on MS data acquisition in the “specific” MaxQuant search mode, by extraction method; 1-Acid, 2-AmBic, 3b-EDTA LysC Neutral, 4a-EDTA Protease Mix Acidic, 4b-EDTA Protease Mix Neutral, 5b-EDTA + GuHCl Neutral, 6-SPIN. (**a**) The number of MS spectra recorded in each raw file per specimen, (**b**) the number of MS/MS spectra recorded in the raw files acquired per specimen, (**c**) the total number of identified tandem MS spectra, and (**d**) the ratio of identified tandem MS spectra in comparison to SPIN for each specimen. In (**d**), the dashed line represents extraction method 6 (value = 1). Extraction methods 3a-EDTA LysC Acidic and 5a-EDTA + GuHCl Acidic were excluded from the study as no reliable MS spectra were generated.

Optimized LC–MS/MS analysis aims to strike a balance between MS1 cycle times and MS2 spectral acquisition rates. We observed that, regardless of extraction method, the GdC samples generated more acquired MS1 scans than Ranis (F = 153.68, p < 2.2e − 16; Fig. 2a). For Ranis, extraction approach 6 generated approximately 2500–4500 MS1 scans, while all other extraction methods obtained approximately 2500 MS1 scans each. For GdC, extraction approach 4a generated the highest number of MS1 scans (> 6000) while the remaining extraction methods acquired approximately 2500–6000 MS1 scans, with generally a high variability between specimens (F = 6.95, p < 9.452e − 06). These observations are evidence of the generally lower proteome preservation in the GdC specimens.

In contrast to MS1, a reverse pattern was obtained for MS2 spectral acquisition (Fig. 2b). Here, the acquired MS2 scan numbers were higher for the Ranis specimens than GdC specimens (F = 161.19, p < 2e − 16). For Ranis specimens, all extraction methods except method 6 resulted in the acquisition of approximately 20,000 MS2 scans. Method 6 resulted in 12,000–17,000 MS2 spectra. For GdC specimens, MS2 acquisition varied. The highest number of MS2 scans (approximately 5500–20,000) was obtained with extraction approach 1, while method 5b generated approximately 5000–18,000 MS2 scans (F = 6.60, p < 1.7e − 05).

We found clear differences in MS2 spectral identification between Ranis and GdC bone extracts for all extraction methods (F = 307.73, p < 2e − 16) (Fig. 2c). These observations also extend to the “semi-specific” search results (Supplementary Fig. S3, SI). There was an overlap in identified MS2 counts between extraction methods. We found that extraction approach 1 generated the highest number of identified MS2 scans for Ranis bone specimens (approximately 500–1500). Identified MS2 scans with extraction method 4, both in 4a and 4b, showed a higher consistency among Ranis specimens (> 900 MS2 identified scans). For GdC specimens, extraction protocol 1 was the only approach resulting in over 500 identified MS2 scans (for four out of six specimens from GdC). All the remaining proteomic extraction methods recovered less than 300 identified MS2 scans for GdC specimens. We also observed significant diffences for the average ion intensity among all the extraction methods for both archaeological sites (Supplementary Fig. S4, SI) in the “specific” (F = 596.09, p < 2.2e − 16) and in the “semi-specific” search (F = 454.58, p < 2.2e − 16).

Finally, we compared the number of identified MS2 scans obtained with extraction method 6 to the other extraction methods (F = 3.08, p = 0.010; Fig. 2d). For Ranis specimens, we observed that extraction method 4, in both 4a and 4b conditions, generated more identified MS2 scans for several specimens compared to method 6. The remaining extraction approaches showed no significant differences in the identified MS2 scans compared to method 6. For GdC specimens, extraction protocol 1 obtained a higher ratio of identified MS2 spectra compared to method 6, while all other approaches resulted in a lower MS2 identification ratio.

### Proteome composition and species identification

**Figure 3:**
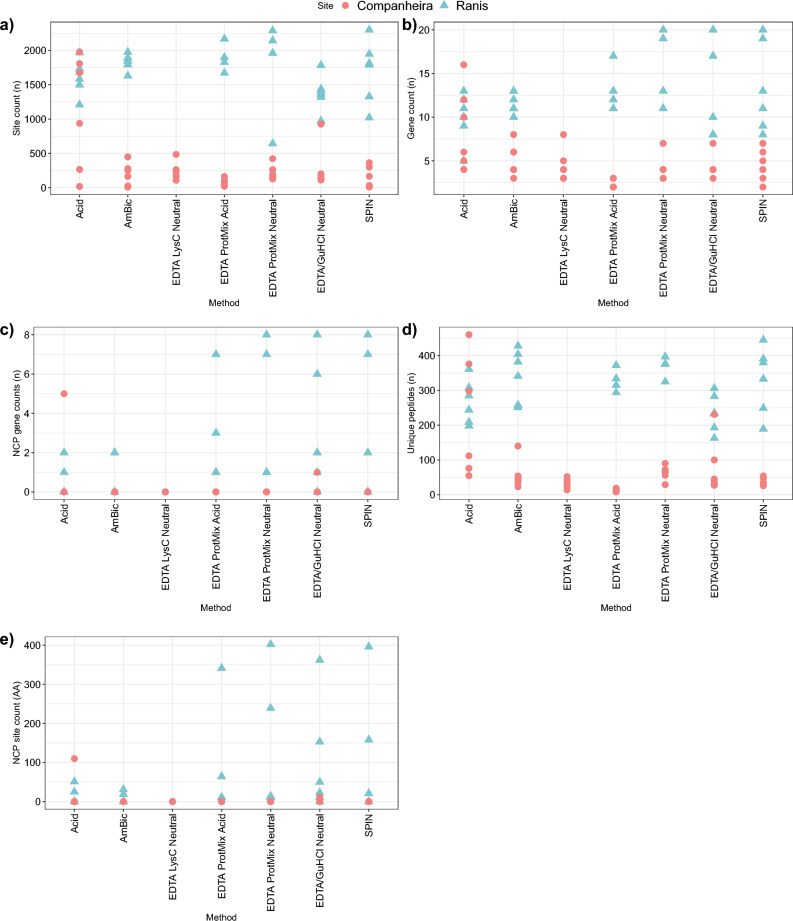
Proteome composition and peptide recovery for all the specimens by extraction method in the “specific” MaxQuant search; 1-Acid, 2-AmBic, 3b-EDTA LysC Neutral, 4a-EDTA Protease Mix Acidic, 4b-EDTA Protease Mix Neutral, 5b-EDTA + GuHCl Neutral, 6-SPIN. (**a**) Number of amino acid positions identified for species identification. (**b**) Number of identified protein genes for each specimen. (**c**) Number of non-collagenous proteins identified for each specimen. (**d**) Number of unique peptide sequences recovered for each specimen. (**e**) Number of amino acids for non-collagenous proteins recovered for each specimen. Extraction methods 3a-EDTA LysC Acidic and 5a-EDTA + GuHCl Acidic were excluded from the study as no reliable MS spectra were generated.

The SPIN identification approach^3^ focuses on 20 protein-coding genes and uses gene-wise sequence coverage estimation for species identification. To further investigate the efficiency of extraction methods to recover peptides belonging to non-collagenous proteins (NCPs), we compared the protein sequence coverage (site counts)^3^ for each specimen. Most of the covered sequences were concentrated in the two collagen type I chains for all the specimens. The number of identified amino acids decreased in all GdC specimens and the majority of the methods generated no amino acid sequence coverage of non-collagenous proteins for GdC specimens (Fig. 3 and Supplementary Table S1, SI). Extraction approach 1 generated a high number of amino acid site counts for most of the GdC specimens, while extraction approaches 4b and 6 had the highest site counts for Ranis specimens. Non-matching species-specific site counts (amino acid sequences for different taxa or closely related species) were below 500 apart from one specimen with approach 3b (not shown). In agreement with the site counts, extraction methods 4b and 6 obtained the highest number of gene counts, NCP gene counts, unique peptides, and NCP site count for Ranis specimens, while extraction approach 1 showed the best results for the GdC specimens.

**Figure 4:**
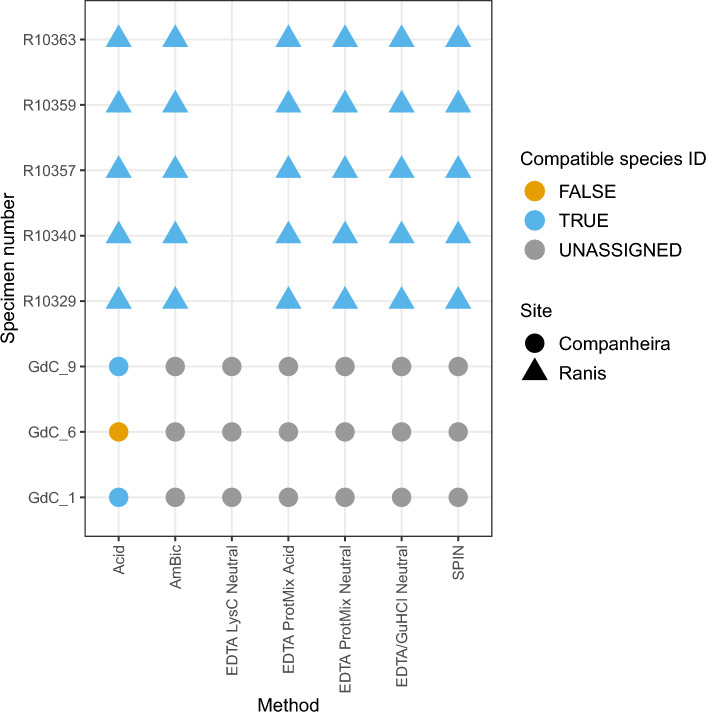
Compatibility of species identification in the “specific” MaxQuant search per specimen for each protein extraction approach; 1-Acid, 2-AmBic, 3b-EDTA LysC Neutral, 4a-EDTA Protease Mix Acidic, 4b-EDTA Protease Mix Neutral, 5b-EDTA + GuHCl Neutral, 6-SPIN. “Unassigned” specimens did not generate reliable results for species identification compared to their previous taxonomic identities. Of the 12 analyzed specimens in this study, specimens “GdC_7”, “GdC_26” and “GdC_193” were excluded from this table because there was no precise species identification assigned previously. “Ra_10300″ was also excluded from the table because no member of the Rhinocerotidae family is included in the Rüther et al. 2022^3^ reference protein sequence database. Extraction methods 3a-EDTA LysC Acidic and 5a-EDTA + GuHCl Acidic were excluded from the study as no reliable MS spectra were generated.

We also compared the species identification provided for the same specimen per extraction method (Fig. 4 and Supplementary Table S1, SI). Ranis specimens generated compatible species identification for all of the extraction methods apart from extraction approach 3b, which gave no data for all Ranis specimens. In general, most of GdC specimens were not identified; only two specimens (Gdc_1 and GdC_9) were correctly assigned to the same species as previously identified, while GdC_6 was falsely assigned with extraction approach 1.

### Peptide characteristics

**Figure 5:**
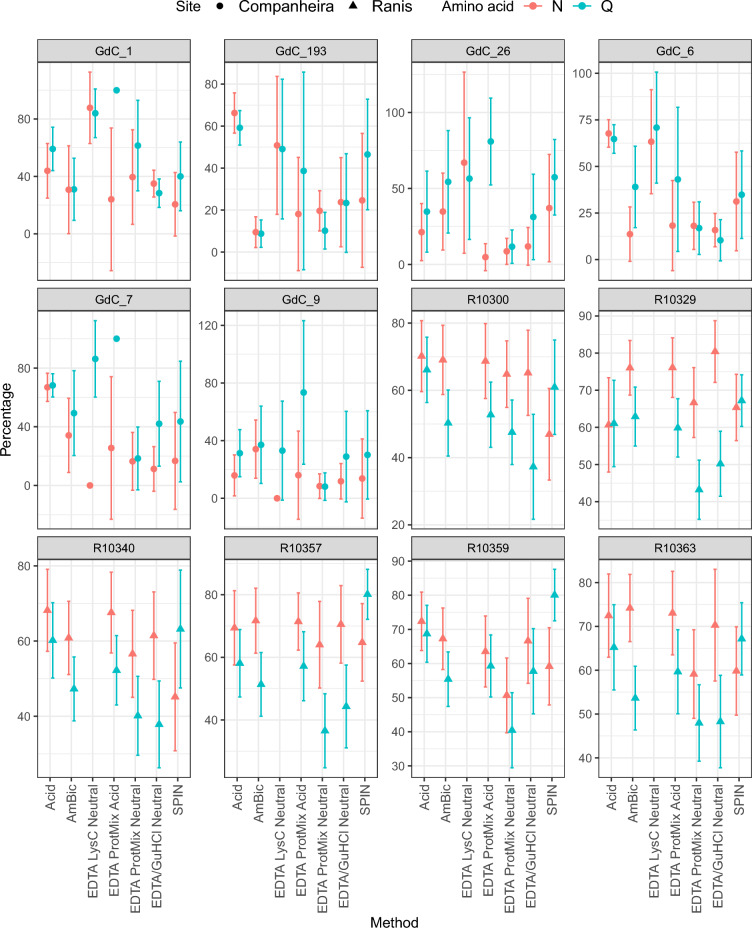
Amino acid deamidation in the “specific” MaxQuant search for each sample by extraction method; 1-Acid extraction, 2-AmBic, 3b-EDTA LysC Neutral, 4a-EDTA Protease Mix Acidic, 4b-EDTA Protease Mix Neutral, 5b-EDTA + GuHCl Neutral, 6-SPIN. Glutamine (Q) and asparagine (N) deamidation for each sample by extraction method. The y-axis represents the percentage of deamidation rate for Q and N, where 100% indicates complete deamidation and 0% indicates no deamidation. Error bars represent 2SD. EDTA LysC Neutral (3b) extraction method generated no results for Ranis specimens. Extraction methods 3a-EDTA LysC Acidic and 5a-EDTA + GuHCl Acidic were also excluded from the study as no reliable MS spectra were generated.

Over time, ancient proteomes are modified in various ways due to diagenesis, such as protein deamidation and peptide bond cleavage^48^. Therefore, we explored differences in the estimated extent of deamidation and semi-specific cleavage rates in our extracts to quantify the effect different extraction protocols may have on these variables. As all extraction methods were performed on the same homogenized bone powders, there should be no significant differences in the obtained values between the extraction methods. Our analysis of deamidation rates was complicated due to the low-quality spectra generated for GdC specimens (F = 2.8206, p = 0.01746). For Ranis specimens, we observed generally consistent rates of glutamine and asparagine deamidation across the neutral extraction methods and slightly higher deamidation values for acidic demineralization methods (Fig. 5 and Supplementary Fig. S5, SI, excluding method 6).

Mis-cleaved peptides for each extraction method per site were also estimated to validate our results for both searches (specific and semi-specific). The specific search was set up to 2 mis-cleaved peptides while the semi-specific search was up to 4 mis-cleavages for both archaeological sites by MaxQuant default parameters (Supplementary Figs. S7 and S8, SI). Extraction approach 3a is excluded from this comparison because LysC only cleaves at lysine (K) sites of the protein sequence. In the specific search, methods 5b and 1 had the highest rate of mis-cleavages for Ranis and GdC specimens accordingly, while method 6 had the lowest for both sample sets apart from extraction method 4a. In the semi-specific search, methods 5b and 6 had the highest rate of mis-cleavages for Ranis and GdC specimens accordingly.

The distribution of peptides according to their hydrophobicity (as expressed by the grand average of hydropathy GRAVY) scale using the method of Kyte and Doolittle^49^) was evaluated for all proteomic extraction methods (Supplementary Fig. S9, SI). The average GRAVY scores for all extraction methods are below 0, indicating a higher number of hydrophilic peptides. The hydrophobicity scores for Ranis specimens were not significantly different for all extraction methods, while GdC specimens showed different values of hydrophobicity depending on the extraction method (F = 28.16, p = 1.247e − 06). The extraction methods 1, 2, and 3b seemed to cluster together and 4a, 4b, 5b, and 6 were grouped together in a higher hydropathy score (F = 6.31, p = 2.421e − 05).

## Discussion and conclusion

Archaeological and palaeontological skeletal remains are a limited resource but contain an extensive amount of information about the past. The development of high-throughput shotgun proteomics (HTS) approaches allows us to identify skeletal remains of otherwise scarce animal species, such as hominins. Hence, optimizing methods of skeletal proteome extraction from small sample sizes with poor preservation becomes relevant. We, therefore, compared six proteomic extraction approaches on Late Pleistocene (LP) remains with variable proteomic preservation.

Different extraction approaches yielded the highest number of identified peptides based on the preservation condition of the specimens. Even though we were able to retrieve more species-specific amino acid sequences with the acidic extraction approach, we acquired valid species identifications for only two specimens from GdC. For Ranis specimens, extraction method 4a generated the highest number of identified peptides, which is in accordance with previous studies^50^. In contrast, extraction method 1 outperformed the other methods for GdC samples. Even though an acidic extraction environment might negatively affect peptide fragmentation and MS2 intensity, we recovered a higher number of unique identified peptides and valid species identifications in acidic environments. This might be explained based on acidic demineralization solutions resulting in proteins being more effectively released from the bone skeletal matrix in association with the removal of protease inhibitors, in contrast with neutral solutions^46^.

The GdC specimens generated a higher number of MS1 scans and a lower number of MS2 scans than the Ranis specimens by all extraction methods. Hence, Ranis specimens generated more MS2-identified peptides than poorly preserved bone specimens. This difference in MS2-identified scans is because, in DDA mode, GdC specimens do not trigger MS2 scan events as frequently as the minimum ion intensity threshold is only irregularly reached, probably because of highly fragmented peptides based on the preservation of the specimens.

In ideal conditions, deamidation per specimen is expected to show the same value regardless of the extraction method, as the subsamples are derived from a single, homogenized bone powder^30,51,52^. Nonetheless, this pattern was not shown in our set of specimens. Therefore, our study demonstrates that protein extraction conditions have variable impacts on glutamine and asparagine deamidation. Due to differences in preservation, we observed a variety of deamidation values between the two sets of specimens. However, our estimates of deamidation rates for the GdC specimens cannot be compared properly due to the low number of identified MS2 spectra. The literature on relative glutamine (Q) and asparagine (N) deamidation rates indicates that glutamine residues deamidate at a slower rate than asparagine residues^53^, as observed in previous palaeoproteomic studies^5^. It is therefore of note that, in our study, we observed that asparagine deamidation ratios for method 6 are below those obtained for glutamine. This pattern, which is the reverse of theoretical expectations, as well as the reverse of most experimentally observed datasets, is difficult to explain. It is, however, consistently present among the Ranis specimens, possibly in the GdC extracts too, and extends to the semi-tryptic search (Supplementary Fig. S10, SI). We therefore believe the observed pattern of more advanced deamidation for glutamines compared to asparagines might be due to the reduction/alkylation step unique to the SPIN protocol in our comparison. Further work in the palaeoproteomics research community should explore this phenomenon in other extraction methods and sample contexts.

In our study, proteomic characteristics were evaluated, such as cleavage rates, NCP genes, and hydropathy, to ensure that the extraction methods do not introduce biases into the recovered proteome. The cleavage rate varied between the extraction methods and sample sets in the specific search. The most abundant identified peptides were assigned to collagen. Most of the extraction methods identified no NCPs in any of the specimens. The pattern of NCPs in the Ranis specimens might be explained based on the nature of each protocol. Novel extraction approaches for ZooMS focus on capturing collagenous proteins due to the specificity of the technique, while SPIN was initially designed to retrieve 20 collagenous and non-collagenous protein-coding genes. Additionally, the hydrophobicity of the peptide extracts was calculated as the average of the hydropathy of the identified amino acids^54^. Based on the difference in identified peptide intensity, it is shown that different amounts of hydrophilic peptides were extracted by each extraction method. Extraction methods 1, 2, and 3b indicated a bias towards hydrophilic peptides, while 4a, 4b, 5b, and 6 indicated a bias towards hydrophobic peptides. In particular, SPIN showed a significant bias towards hydrophobic peptides. This was probably due to the PAC extraction step, as magnetic sulfur beads tend to capture hydrophobic proteins based on the chemical characteristics of the ligand on the surface of the microbeads.

We presented a comparison of destructive proteomic extraction approaches for species identification by LC–MS/MS. Our results showed that preservation conditions should be taken into consideration when designing proteomic extractions from archaeological bone specimens. Therefore, a pilot study that compares a few extraction approaches based on sample preservation is advised for optimal results. We demonstrated that the acid protein extraction method produces high-quality spectra for bone proteome analysis by LC–MS/MS for degraded samples, would allow for an easy scale-up, and could be preceded by ammonium bicarbonate buffer extraction method, as performed in several ZooMS studies^7,17,18^. For degraded specimens, we also suggest loading a higher amount (over 10%) of the resulting peptide solution, to enhance MS2 acquisition. An additional step measuring protein concentration in several samples before Evotip loading might be beneficial to calculate the necessary amount of peptides for optimal LC–MS/MS runs. Moreover, especially in challenging contexts such as the Late Pleistocene, our study showed that the adjustment of data analysis should be considered when designing HTS proteomic studies from archaeological bone specimens. A thorough understanding of the impacts of laboratory processing protocols and analysis methods on the reconstructed proteome is thereby essential to retrieve a maximum amount of unbiased proteomic information from archaeological specimens.

## Methods

### Specimen selection and sampling

We randomly selected a total of 12 morphologically unidentified bone specimens from two different archaeological sites (Table 1 and Supplementary Fig. S1, SI); six bone specimens from GdC (Portugal) and six bone specimens from Ranis (Germany). Both sets of archaeological specimens date to the Late Pleistocene (LP) and derived from cave sites, with those from GdC expected to be older^3^ (> 50 kya) and preserved at comparatively high, Mediterranean temperature conditions. GdC is situated on a hill at a height of 20 m above the current sea level, primarily composed of Jurassic and Dolomitic limestones. In contrast, Ranis is slightly younger (unpublished data; 40–50 kya) with comparatively low, central European temperature conditions. The Ranis bone specimens come from the Layer X (Graue Schicht) of the 1932–1938 excavation^55^. This layer is a dark grey humic loamy silt formed in a cave sedimentary environment. The archaeological and paleontological material represents a mix of bone accumulations by carnivores as well as by short-term human occupations. The GdC bone specimens derive from Chamber 2, where the materials used in this study were recovered at the base of a loose sediment talus. The infill is composed of calcareous sediment, limestone blocks collapsed from the cave ceiling, terra rossa, Middle Palaeolithic stone tools, as well as faunal remains^56^. Specimens from GdC displayed variable but generally poor proteome recovery in previous SPIN analysis^3^, while collagen preservation and ZooMS identification rates at Ranis indicate well-preserved skeletal proteomes (unpublished results). These prior analyses also provided some taxonomic information for most of the specimen, despite all 12 bone specimens being unidentifiable based on morphological characteristics (Table 1).

Sampling took place in a flow box at the Max Planck Institute for Evolutionary Anthropology (Leipzig, Germany). Surfaces were covered with aluminium foil, which was replaced after sampling each specimen. The flow box, the drilling equipment, and other utensils were cleaned with 70% ethanol and air-dried before use. The samples were drilled into a fine powder using a dental drill. Drill heads were sonicated in 70% ethanol for 10–15 min and air-dried before every use. Approximately 100 mg of bone powder for each sample was generated and homogenized. Subsequently, 5 mg of each sample was placed into a clean 1.5 ml Lo-bind protein Eppendorf tube for each extraction. The remaining bone powder was stored in a separate clean 1.5 ml Lo-bind protein Eppendorf tube at room temperature for potential future analysis.

### Proteome extraction methods

We compared the proteome recovery of six proteomic extraction methods (Fig. 1 and Supplementary Table S1, SI). Proteome extraction procedures took place at the Center of Protein Research (Copenhagen, Denmark), where standard laboratory procedures minimized the risk of modern contamination. We assessed two simple methods broadly used for ZooMS, which use ammonium bicarbonate (AmBic or ABC)^42^ and a conventional acid demineralization extraction method^4^. Additionally, two EDTA-based methods with modifications in the digestion enzyme^5,43,44,57^ were tested in this study. We also included a digestion protocol only with LysC. Even though it is commonly used in modern proteomics^58^, studies of ancient proteins demonstrate that these proteins are frequently hydrolyzed to fragment sizes smaller than those encountered in modern proteins^59^. Therefore, the digestion using LysC and trypsin sequentially could, theoretically, lead to an over-cleavage of the surviving protein fragments. Finally, a PAC extraction method^3^ and an EDTA-based method that combines demineralization and denaturation steps followed by protein digestion in situ^45,46^ were also compared.

Here, in situ digestion refers to a digestion where both the insoluble bone pellet, left after denaturalization, and the soluble protein solution, after denaturation, are still present. In solution digestion refers to a digestion of proteins in solution after denaturalization only. A brief description of each extraction protocol is given below, whilst all materials and equipment used for this study are shown in Supplementary Table S2, SI. Negative controls were included for each extraction method.

1. Acid-in solution digestion. Samples were demineralized with 100 µL 5% HCl overnight, at RT. Demineralized samples were centrifuged at 10,000 × *g* for 10 min and the supernatant was discarded. The acid-insoluble residue was then washed 3 times with 100 µL 50 mM ammonium bicarbonate and the pH was ensured to be at 8.0 using pH paper sticks. Then, samples were denatured at 65 °C in 100 µL 50 mM ammonium bicarbonate for 1-h incubation. Following denaturation, the samples were centrifuged at 10,000 × *g* for 10 min to precipitate ungelatinized protein. Fifty μL of the supernatant was transferred to a clean 96-well plate and the samples were digested with 1 μL of 50 mM trypsin solution overnight at 37 °C. After digestion, samples were centrifuged at 10,000 × *g* for 1 min and acidified using 5 μL of 5% TFA. The samples were centrifuged at 10,000 × *g* for 1 min and the supernatant was transferred to Evotips for peptide purification and LC–MS/MS analysis.
2. Ammonium bicarbonate- in solution digestion. Five mg of homogenized bone powder was suspended and denatured in 100 µL 50 mM ammonium bicarbonate pH 8.0 at 65 °C for 1 h incubation. Following denaturation, the samples were centrifuged at 3,000 × *g* for 10 min. Fifty μL of the supernatant was transferred to a clean 96-well plate and digested with 1 μL of 50 mM trypsin solution overnight at 37^ο^C. After digestion, samples were centrifuged at 10,000 × *g* for 10 min and acidified using 5 μL of 5% TFA. The samples were centrifuged at 3000 × *g* for 5 min and the supernatant was transferred to Evotips for peptide purification and LC–MS/MS analysis.
3. EDTA LysC- in situ digestion. The samples were suspended in 100 μL 0.5 M EDTA pH 8.0 and incubated overnight, at RT with gentle agitation on a shaker. After demineralization, 1 μL of 50 mM LysC was added to each sample. The samples were digested overnight, at 37 °C with gentle agitation on a thermoshaker. After centrifugation at 10,000 × *g* for 10 min, half of the supernatant was acidified with 5 μL of 5% TFA, and the supernatant was transferred to a new Stagetip (3a, excluded from the study), and the other half was transferred to a Stagetip (3b). After Stagetip clean-up, the peptides were loaded to separate Evotips for LC–MS/MS analysis.
4. EDTA Protease mix- in situ digestion. Samples were suspended in 100 μL 0.5 M EDTA pH 8.8 and incubated overnight, at RT with gentle agitation on a thermoshaker. After demineralization, the samples were removed from the thermoshaker and 3 μL of protease mix LysC/Trypsin (1:2) was added to each sample. The samples were digested overnight, at 37 °C with gentle agitation. After centrifugation at 10,000 × *g* for 10 min, half of the supernatant was acidified with 5 μL of 5% TFA and the supernatant was transferred to a new Stagetip (4a), and the other half was transferred to a Stagetip (4b). After Stagetip clean-up, the peptides were loaded to separate Evotips for LC–MS/MS analysis.
5. EDTA + GuHCl- in situ digestion. The samples were demineralized and denatured with 600 µL solution of 0.5 M EDTA and 3 M GuHCl overnight, at RT. The demineralized samples were centrifuged at 10,000 × *g* for 10 min and the supernatant was discarded. The acid-insoluble residue was washed with 100 µL 50 mM ammonium bicarbonate until the pH was at 8.0. 175 μL of 50 mM ammonium bicarbonate and 1.5 μL of protease mix of LysC/Trypsin were added to the sample. After centrifugation at 10,000 × *g* for 10 min, half of the supernatant was acidified with 5 μL of 5% TFA, and the supernatant was transferred to a new Stagetip (5a, excluded from the study), and the other half was transferred to a Stagetip (5b). After Stagetip clean-up, the peptides were loaded to separate Evotips for LC–MS/MS analysis.
6. SPIN- in solution digestion. The samples were suspended in 100 µL 5% HCl and 0.1% NP-40 solution for overnight demineralization at RT with continuous shaking at 1000 rpm. Reduction, alkylation, and collagen denaturation were facilitated by adding 10 µL 0.1 M tris(2-carboxyethyl) phosphine (TCEP) and 0.2 M N-ethylmaleimide (NEM) in 50% ethanol and 50% ultrapure water and shaking at 1000 rpm at 60 °C, for 1 h. The purification and digestion took place on a KingFisherTM Flex (ThermoFisher Scientific) magnetic bead-handling robot. Debris was removed from the protein extract by centrifuging the plate at 800 × *g*, for 5 min. Magnetic SiMAG-Sulfon beads were washed and prepared at a final concentration of 5 mg/mL in 60% ACN. In a deep-well KingFisherTM plate, 10 µL bead solution and 40 µL of the clear protein extract were briefly mixed. Protein aggregation capture (PAC) was initiated by the addition of 240 µL 70% acetonitrile (ACN; 60% final concentration). The robot was loaded with plates “wash I” (500 µL 70% acetonitrile, 30% water), “wash II” (500 µL 80% ethanol, 20% water), “wash III” (500 µL 100% acetonitrile, and the “on-bead-digestion” plate (100 µL 20 mM Tris pH 8.5, 1 µg/mL LysC, 2 µg/mL Trypsin). The programmed sequence was: (i) collect the beads with low speed for 3:30 min, (ii–iv) wash I-III with slow mixing for 2 min, and (v) bead release on the digestion plate. The digestion was finalized outside the robot, shaking at 800 rpm at 37 °C, overnight. The peptides were acidified with 10 µL 5% trifluoroacetic acid (TFA). After acidification, the peptides were purified in an Evotip for LC–MS/MS analysis.

### Peptide purification and Evotip loading

The purification of the digested peptides was performed for all non-EDTA-based extraction methods directly purified on Evotips as described below (methods 1, 2, and 6). However, EDTA in acidic conditions (methods 3, 4, and 5) usually results in precipitates and might block the Evotips, negatively affecting downstream peptide elution and data acquisition during mass spectrometry. To minimize the chances of this happening, all EDTA peptide digestions were split in half and eluted on homemade Stagetips as follows, before Evotip loading. The other half was purified directly to the Evotips. The extraction approaches 3a and 5a generated no MS data and they are excluded from this study.

- *Stagetip equilibration and loading*: One Stagetip was activated per sample. All Stagetips were activated with 50 µL 100% ACN and equilibrated twice with 50 µL 5 mM AmBic. The supernatant was removed between all the steps with centrifugation at 700 × *g* for 60 sec. The equilibrated tips were loaded with the supernatant and washed twice with 50 µL 5 mM AmBic, the pH was adjusted by washing twice with 50 µL of 0.1% TFA and the peptides were eluted with 50 µL 40 % ACN and 50 µL 60 % ACN. Finally, ACN was removed from the eluate with a speed vac.
- *Evotip equilibration and loading*: One Evotip (Evosep, EV-2001) per sample was washed in ACN, soaked with isopropyl alcohol, and equilibrated with 0.1% FA in water, according to the manufacturer’s protocol. The equilibrated tips were loaded with 10% peptide solution (out of 100 μL) and washed with 20 µL 0.1% FA before LC-MS/MS.

### LC–MS/MS for palaeoproteomics

LC–MS/MS was carried out using the 60 samples per day (SPD) DDA method of an Evosep One (Evosep, Odense, Denmark^41^) operated with the Evosep plugin (1.4.381.0) in Chronos (4.9.2.0) and an analytical column made in-house using a laser-pulled 16 cm long 150 μm inner diameter capillary packed with 1.9 μm C18 bounded silica particles (ReproSil-Pur, C18-AQ, Dr. Maisch, Germany). The column was mounted on an electrospray source with a column oven set at 60 °C. Peptides were ionized by nano-electrospray at 2 kV and analyzed on an Orbitrap Exploris 480™ (Thermo Fisher Scientific, Bremen, Germany) MS operated with Xcalibur (3.1–4). Full scans ranging from 350 to 1400 m/z were measured at 60 k resolution, 25 ms max. IT, 300% AGC target. The top 8 precursors were selected (30 s dynamic exclusion) for HCD fragmentation with an isolation window of 1.3 m/z and a NCE of 30. The minimum intensity to trigger an MS2 Scan was lowered to 5e4. MS2 scans were acquired at 15 k resolution, 22 ms max. IT, and 200% AGC target. Using such a short gradient DDA approach reduces the cost per sample by an order of magnitude compared to previous LC–MS/MS-based species identification strategies. In addition to higher throughput and lowered analysis costs, short gradients in the EvoSep One allowed for stable storage of the samples on EvoTips, avoiding preparation steps like peptide elution after cleanup, solvent evaporation, and MALDI target plate spotting.

### MaxQuant search

All .raw files were analyzed in MaxQuant version 2.1.1.0 against a reference protein sequence database provided by Ruther et al. 2022^3^. Download dates are available in the MaxQuant “summary.txt” files for ‘specific’ and ‘semi-specific’ searches (PXD042321- ProteomeXchange online repository). Variable modifications were included, such as oxidation (M), deamidation (NQ), Gln- > pyro-Glu, Glu- > pyro-Glu, and proline hydroxylation, whereas NEM-derivatization of Cys was configured as a fixed modification for method 6. Initial searches were run in specific Trypsin/P digestion mode and allowed for up to two miscleavages. All files were searched against the above-mentioned database using the same settings. Up to five variable modifications were allowed. The internal MaxQuant contaminant list was replaced with a custom database^3^. All other settings were left on default. A second search was performed using semi-specific Trypsin/P digestion mode and up to four miscleavages were allowed, with all other settings left identical.

### Data analysis

After spectral identification, data analysis was conducted largely through R (version 4.1.2) in RStudio (version 2022.02.0.0) using the packages tidyverse (version 1.3.1)^60^, seqinr (version 4.2–8), devtools (version 2.4.4), ggpubr (version 0.4.0), data.table (version 1.14.2)^61^, bit64 (version 4.0.5)^62^, ggsci (version 2.9), progressr (version 0.10.0)^63^, gmp (version 0.6–6)^64^, reshape2 (version 1.4.4)^65^ and stringi (version 1.7.6). Deamidation was quantified based on spectral intensities, following Mackie et al.^51^. Hydropathy values (a measure of hydrophobicity) were estimated for recovered proteins using the web application GRAVY Calculator (www.gravy-calculator.de/) and Peptides (version 2.2.4)^66^. Statistics were calculated using the two-way ANOVA (Type II) tests^67^ from carData (version 3.0–5) and car (version 3.1–0)^68^. The map was built with the package maps (version 3.4.1)^69^.

### Supplementary Information

Supplementary Information.

## Data Availability

The raw mass spectrometry proteomics data generated in this study have been deposited to the ProteomeXchange Consortium via the PRIDE^70^ partner repository with the dataset identifier PXD042321.
