# Supplementary material for: Comparing extraction method efficiency for high-throughput palaeoproteomic bone species identification
Source: Sci Rep. 2023 Oct 26;13:18345. doi: 10.1038/s41598-023-44885-y (PMC10603084; doi:10.1038/s41598-023-44885-y)
Supplement: Supplementary file 1 — Supplementary Information. [file 41598_2023_44885_MOESM1_ESM.pdf]

Comparing extraction method efficiency for high-throughput palaeoproteomic bone species identification.

Dorothea Mylopotamitaki[1,2,*], Florian S. Harking[3], Alberto J. Taurozzi[4], Zandra Fagernäs[4], Ricardo M. Godinho[5], Geoff M. Smith[6,2], Marcel Weiss[7,2], Tim Schüler[8], Shannon P. McPherron[9], Harald Meller[10], João Cascalheira[5], Nuno Bicho[5], Jesper V. Olsen[3], Jean-Jacques Hublin[1,2], Frido Welker[4,*]

1 Chaire de Paléoanthropologie, CIRB (UMR 7241–U1050), Collège de France, Paris, France.
2 Max Planck Institute for Evolutionary Anthropology, Leipzig, Germany.
3 Center for Protein Research, University of Copenhagen, Copenhagen, Denmark.
4 Globe Institute, University of Copenhagen, Copenhagen, Denmark.
5 Interdisciplinary Center for Archaeology and Evolution of Human Behaviour, University of Algarve, Faro, Portugal.
6 School of Anthropology and Conservation, University of Kent, Kent, United Kingdom.
7 Institut für Ur- und Frühgeschichte, Friedrich-Alexander-Universität, Erlangen, Germany.
8 Thuringian State Office for the Preservation of Historical Monuments and Archaeology, Weimar, Germany.
9 Department of Human Origins, Max Planck Institute for Evolutionary Anthropology, Leipzig, Germany.
10 State Office for Heritage Management and Archaeology, Saxony-Anhalt—State Museum of Prehistory, Halle (Saale), Germany.

*Corresponding authors. Dorothea Mylopotamitaki: dorothea.mylopotamitaki@palaeoproteomics.org; Frido Welker: frido.welker@sund.ku.dk

Supplementary Information

Supplementary Table S1. Overview of extraction conditions of the six extraction methods compared in this study. A dash indicates the absence of a particular step. No reliable MS spectra were generated for protocols 3a and 5a and consequently, they were excluded from further analysis. In addition, for Ranis specimens, we did not generate reliable data with extraction approach 3b.

Base protocol	**1-Acid**	**2-AmBic**	**3a-EDTA LysC Acidic pH**	**3b-EDTA LysC Neutral pH**	**4a-EDTA Protease Mix Acidic pH**	**4b-EDTA Protease Mix Neutral pH**	**5a-EDTA+ GuHCl Acidic pH**	**5b-EDTA+ GuHCl Neutral pH**	**6-SPIN**
Demineralization	HCl	-	EDTA	EDTA	EDTA	EDTA	EDTA	EDTA	HCl + NP-40
Denaturation	AmBic/ heat	AmBic/ heat	-	-	-	-	GuHCl/ heat	GuHCl/ heat	NEM
Reduction/ alkylation	-	-	-	-	-	-	-	-	TCEP
Dilution	-	-	-	-	-	-	(in AmBic)	(in AmBic)	-
Protein aggregation	-	-	-	-	-	-	-	-	✓
Protein digestion	in solution digestion	in solution digestion	in situ digestion	in situ digestion	in situ digestion	in situ digestion	in situ digestion	in situ digestion	PAC digestion
Digestion enzyme	Trypsin	Trypsin	LysC	LysC	Trypsin LysC	Trypsin LysC	Trypsin LysC	Trypsin LysC	Trypsin LysC
Acidification	TFA	TFA	TFA	-	TFA	-	TFA	-	TFA

Supplementary Table S2. Material and equipment used in the study.

Reagents, Instruments, and Consumables	Company	Catalog number
0.5 M EDTA (Ethylenediaminetetraacetic acid)	Invitrogen™	AM9260G
Trypsin (0.4 µg/µL) (5 x 20 µg)	Promega	V5111
rLys-C, Mass Spec Grade (0.2 µg/µL) (15 µg)	Promega	V1671
Trifluoroacetic acid (TFA)	Sigma-Aldrich	T6508
Acetonitrile (ACN)	Sigma-Aldrich	34851
Hydrochloric acid (HCl) 37% (v/v)	Sigma-Aldrich	H1758
Guanidine Hydrochloride Solution 6M (GuHCl)	Sigma-Aldrich	SRE0066
Ammonium Bicarbonate (AmBic)	Sigma-Aldrich	09830
NP-40 Surfact-Amps ™ Detergent Solution 10% (w/v) (NP-40)	Thermo Scientific™	85124
Tris-HCl pH 8.5 (MW: 121,14 g/mol)	Sigma-Aldrich	10708976001
Isopropyl alcohol HPLC, 99.9%	Sigma-Aldrich	34863
Tris(2-carboxyethyl) phosphine (TCEP) (MW: 286.65 g/mol)	Sigma-Aldrich	C4706
N-ethylmaleimide (NEM) (MW: 125.13 g/mol)	Sigma-Aldrich	E3876
Magnetic SiMAG-Sulfon beads (50 mg/ml)	Chemicell	1202
Ethanol 99.9 % (v/v) (EthOH)	Thermo Scientific™	10542382
UHQ water		
Evotips (96-tip Plate)	Evosep	EV-2003
Stagetips (“Homemade”)		
Chromatography column (“Homemade”)		
Evosep-One	Evosep	EV-1000
Orbitrap Exploris 480	Thermo Fisher Scientific	
KingFisher™ Flex Purification System	Thermo Fisher Scientific	
Centrifuge 5234 Thermo Fisher Scientific	Thermo Fisher Scientific	
Thermomixer C	Thermo Fisher Scientific	
IKA MS 3 Basic Vortexer	Overstock Lab Equipment	
Ultra-Fine Scale	Sartorius Stedim	
96 Well Plates and Plate Seals for Kingfisher	Thermo Fisher Scientific	
96 Deep Well Plates for Kingfisher	Thermo Fisher Scientific	
Protein Lo-Bind tubes (1.5 ml, 2 ml)	Eppendorf	
pH strips 0-14		
Multi and Single channel pipettes 10, 20, 200, 300, 1000 µL and pipette tips		

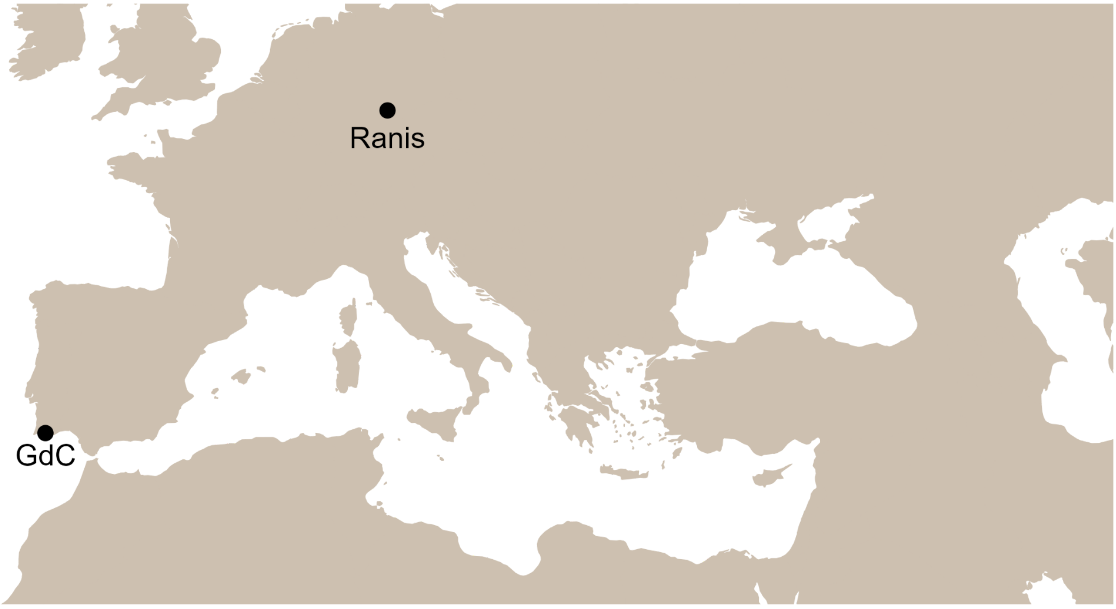

Supplementary Figure S1. World map with both archaeological cave site locations (Ranis, Germany, and Gruta da Companheira (GdC), Portugal). The map was built in (version 4.1.2) https://www.R-project.org/ in RStudio (version 2022.02.0.0) http://www.rstudio.com/ with the package maps (version 3.4.1) https://cran.r-project.org/web/packages/maps/index.html.

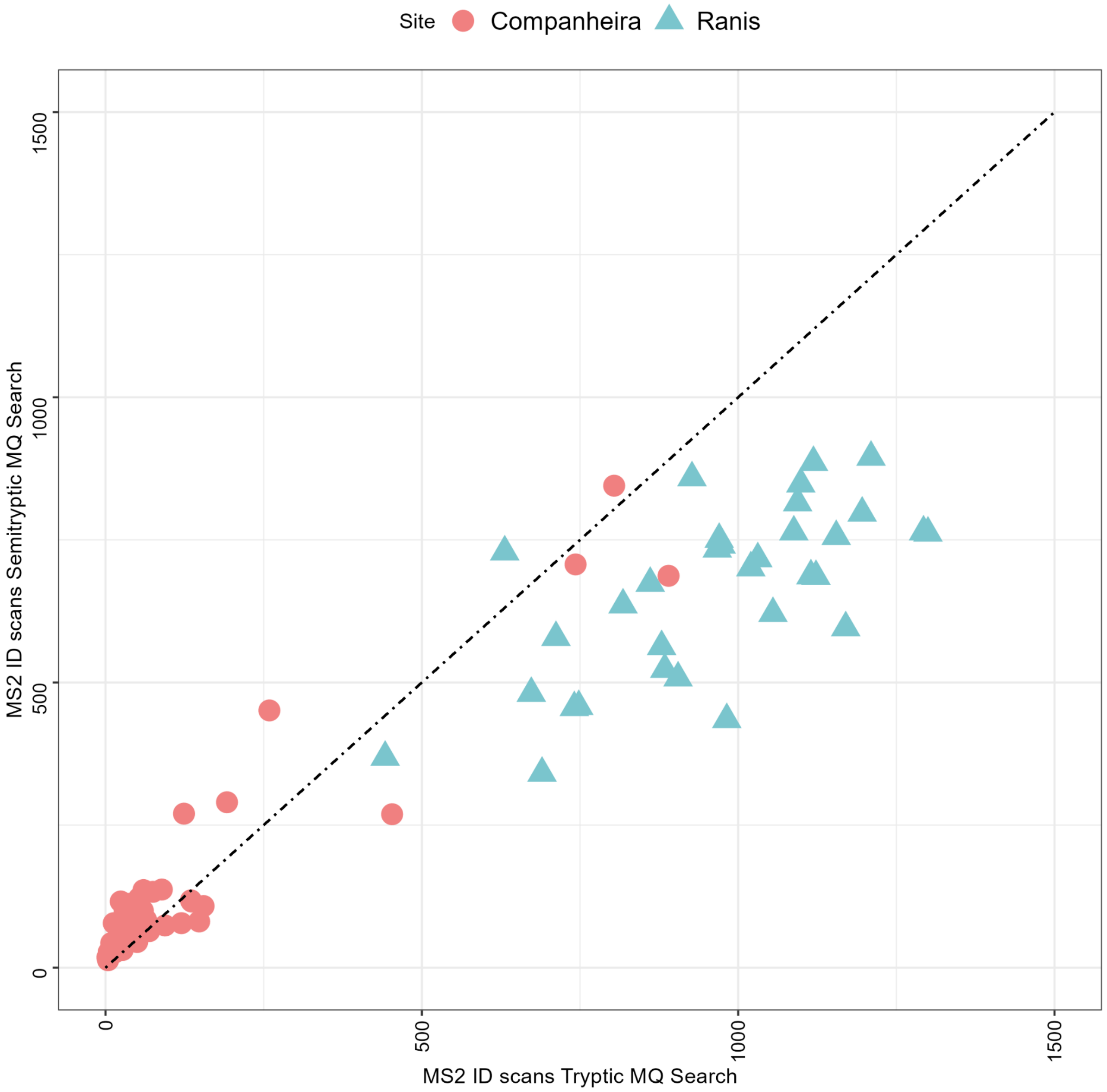

Supplementary Figure S2. Comparison of the number of identified MS2 scans using "specific" and "semi-specific" search settings in MaxQuant. Each point indicates a .raw data file, corresponding to one extraction method condition for a bone specimen. The dashed line equals x=y.

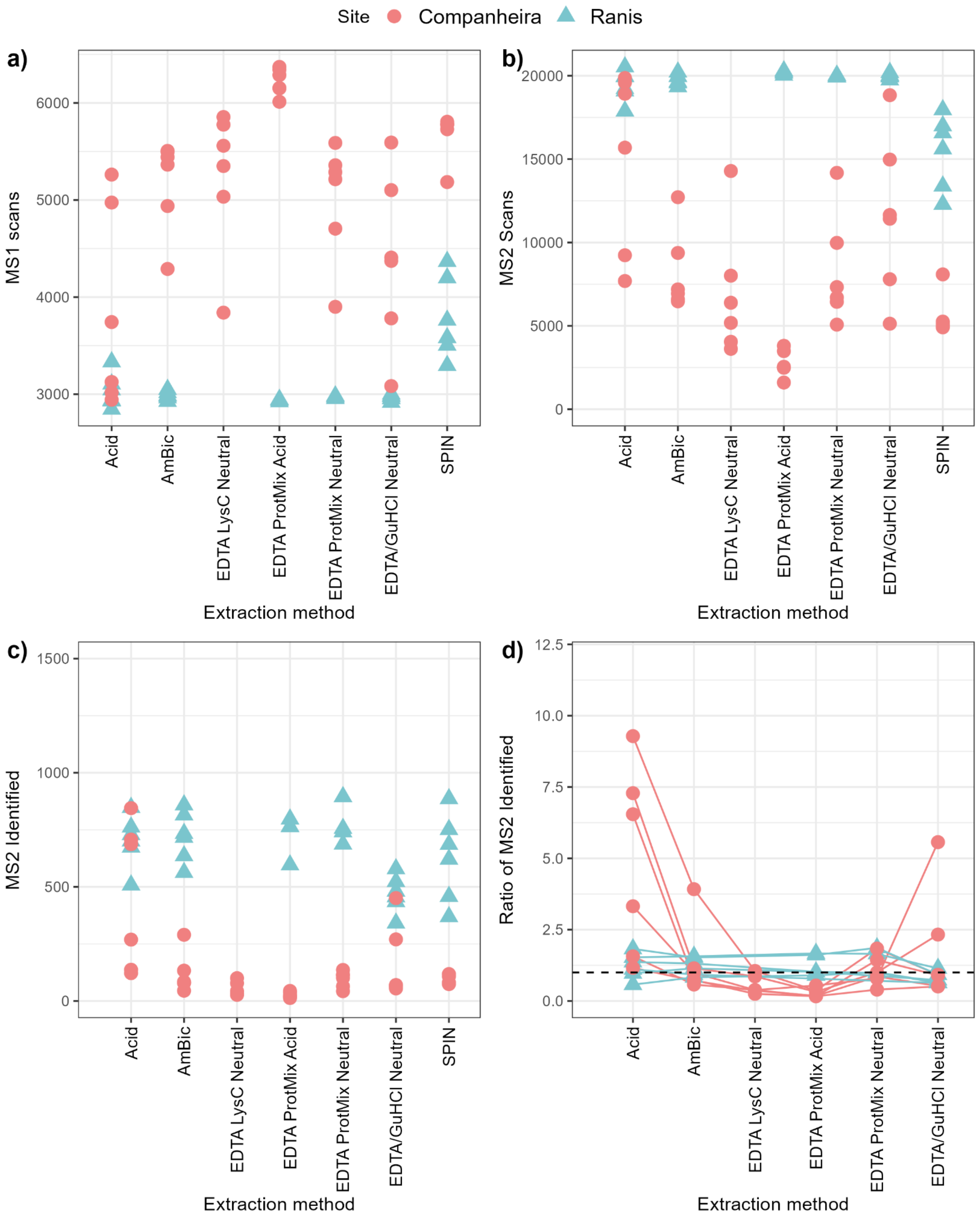

Supplementary Figure S3. Summary information on MS data acquisition in the "semi-specific" MaxQuant search, by extraction method; 1-Acid, 2-AmBic, 3b-EDTA LysC Neutral, 4a-EDTA Protease Mix Acidic, 4b-EDTA Protease Mix Neutral, 5b-EDTA+GuHCl Neutral, 6-SPIN. **a)** The number of MS spectra recorded in each raw file per specimen, **b)** the number of MS/MS spectra recorded in the raw files acquired per specimen, **c)** the total number of identified tandem MS spectra, and **d)** The ratio of identified tandem MS spectra in comparison to SPIN for each specimen. In (d), the dashed line represents extraction method 6 (value = 1). Extraction methods 3a- EDTA LysC Acidic and 5a- EDTA+GuHCl Acidic were excluded from the study as no reliable MS spectra were generated.

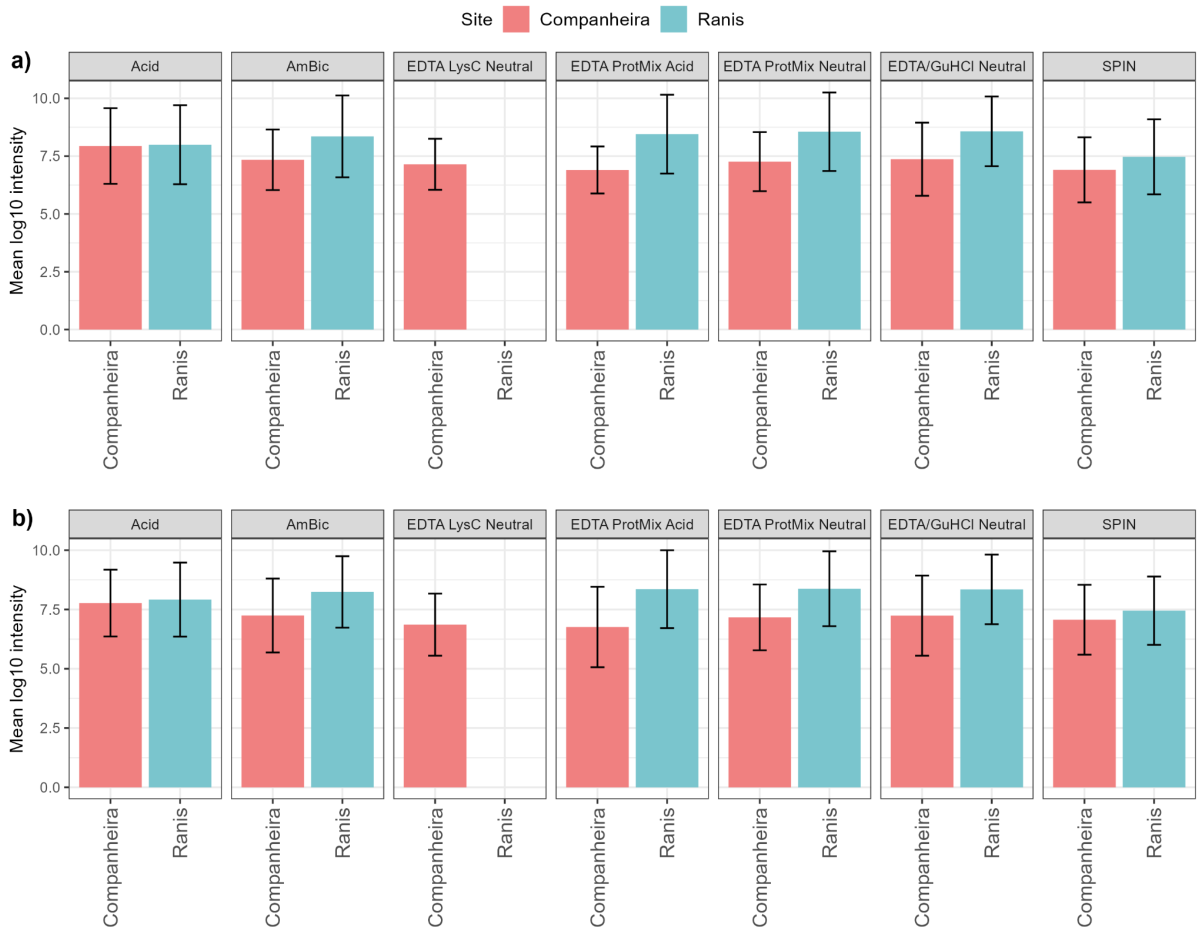

Supplementary Figure S4. Average ion intensity (log10) in the "specific" MaxQuant search per archaeological site for all extraction methods. 1-Acid, 2-AmBic, 3b-EDTA LysC Neutral, 4a-EDTA Protease Mix Acidic, 4b-EDTA Protease Mix Neutral, 5b-EDTA+GuHCl Neutral, 6-SPIN. **a)** "specific" MQ search and **b)** "semi-specific" MQ search. Extraction methods 3a- EDTA LysC Acidic (Ranis and GdC), 5a- EDTA+GuHCl Acidic (Ranis and GdC), and 3b- EDTA LysC Neutral (Ranis) generated no reliable results specimens, so they are not represented in the plots. The y-axis represents the mean log10 ion intensity. Error bars represent 2SD.

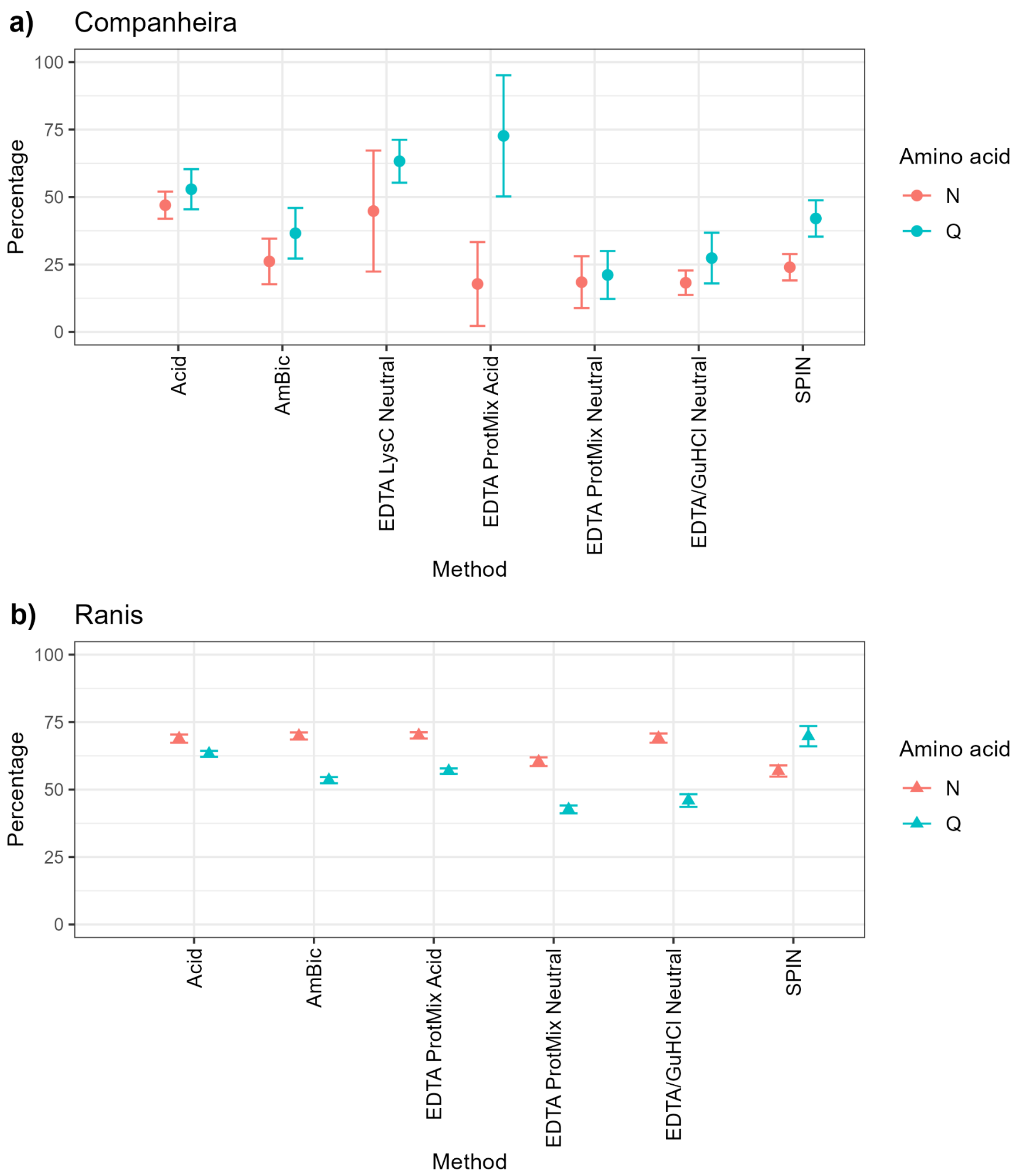

Supplementary Figure S5. Glutamine (Q) and asparagine (N) deamidation for both archaeological sites by all extraction methods, for the "specific" MaxQuant search; 1-Acid, 2-AmBic, 3b-EDTA LysC Neutral, 4a-EDTA Protease Mix Acidic, 4b-EDTA Protease Mix Neutral, 5b-EDTA+GuHCl Neutral, 6-SPIN. Average protein deamidation (Q) and (N) for specific peptides by extraction method **a)** for GdC specimens and **b)** for Ranis specimens. EDTA LysC Neutral (3b) extraction method generated no results so it is not represented in the (b) plot. Extraction methods 3a- EDTA LysC Acidic and 5a- EDTA+GuHCl Acidic were also excluded from the study as no reliable MS spectra were generated. The y-axis represents the percentage of deamidation rate for Q and N, where 100% indicates complete deamidation and 0% indicates no deamidation. Error bars represent 2SD.

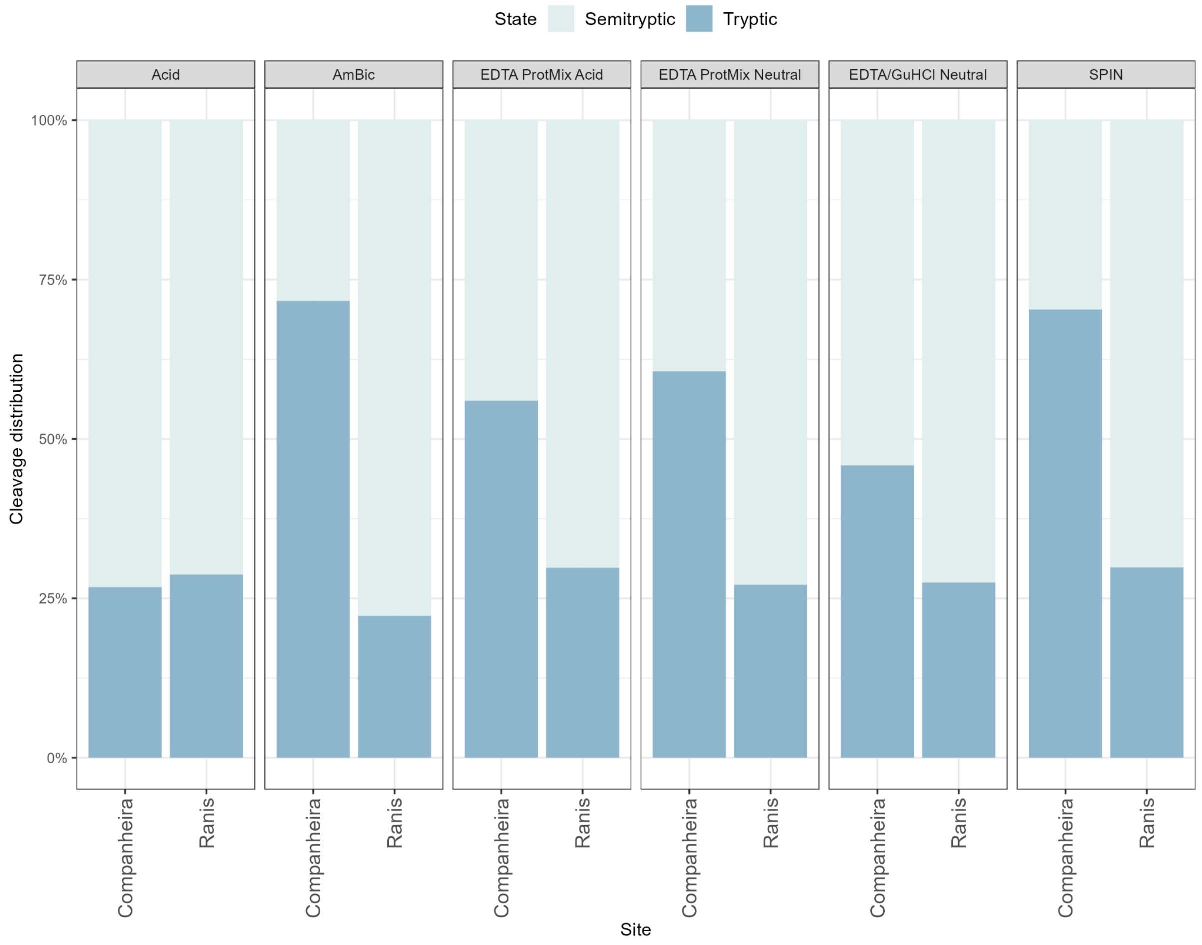

Supplementary Figure S6. Proportions of tryptic and semi-tryptic peptides resulting from the "semi-specific" MaxQuant search, by extraction method for both excavation sites; 1-Acid, 2-AmBic, 3b-EDTA LysC Neutral, 4a-EDTA Protease Mix Acidic, 4b-EDTA Protease Mix Neutral, 5b-EDTA+GuHCl Neutral, 6-SPIN. The y-axis represents the percentage of cleavage rate for each excavation site per extraction method. Extraction method 3b was excluded from this study because LysC cleaves only at K sites. Extraction methods 3a- EDTA LysC Acidic and 5a- EDTA+GuHCl Acidic were also excluded from the study as no reliable MS spectra were generated. With the exception of method 1 (Acid), peptides with semi-tryptic termini are more abundant for well-preserved specimens (Ranis) and dominant for all datasets (approximately 75%). In contrast, the proportion of peptides with semi-tryptic termini varies by extraction method for the degraded specimens (GdC).

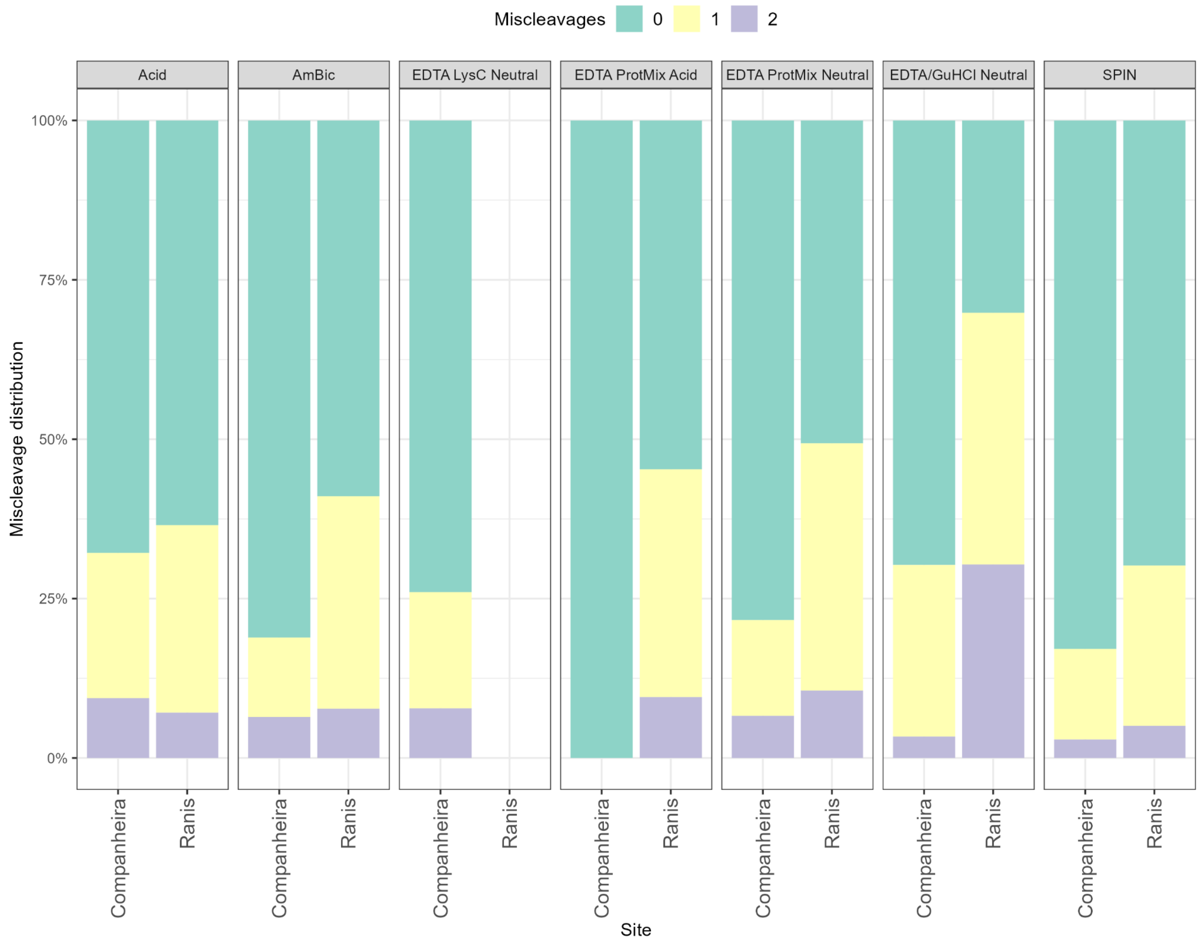

Supplementary Figure S7. Mis-cleavaged peptide proportions for the "specific" MaxQuant search by extraction method for both excavation sites, for Gruta da Compaheira and Ranis specimens; 1-Acid, 2-AmBic, 3b-EDTA LysC Neutral, 4a-EDTA Protease Mix Acidic, 4b-EDTA Protease Mix Neutral, 5b-EDTA+GuHCl Neutral, 6-SPIN. The y-axis represents the percentage distribution of miscleavages for each excavation site per extraction method. Extraction methods 3a- EDTA LysC Acidic and 5a- EDTA+GuHCl Acidic were excluded from the study as no reliable MS spectra were generated.

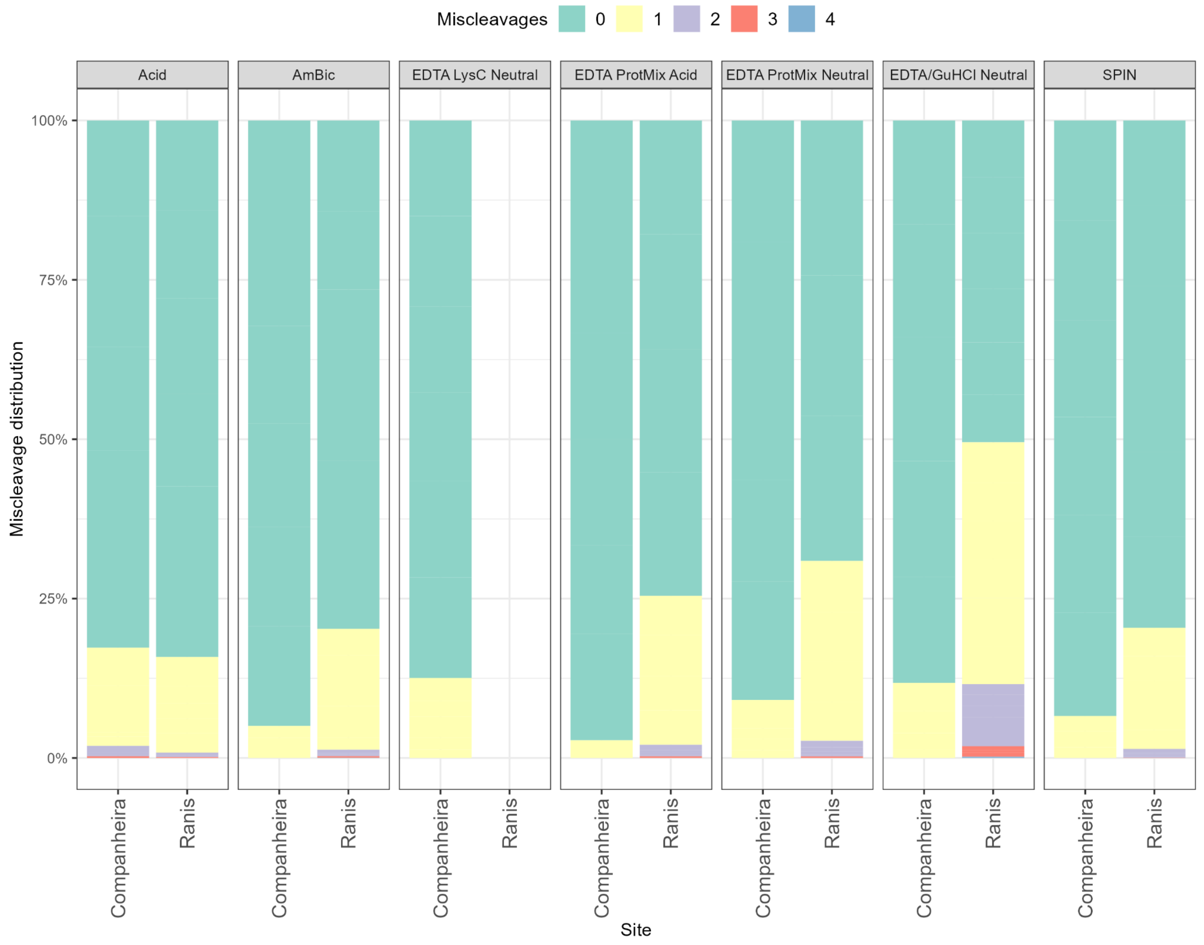

Supplementary Figure S8. Mis-cleavaged peptide proportions for the "semi-specific" MaxQuant search by extraction method for both excavation sites, for Gruta da Compaheira and Ranis specimens; 1-Acid, 2-AmBic, 3b-EDTA LysC Neutral, 4a-EDTA Protease Mix Acidic, 4b-EDTA Protease Mix Neutral, 5b-EDTA+GuHCl Neutral, 6-SPIN. The y-axis represents the percentage distribution of miscleavages for each excavation site per extraction method. Extraction methods 3a- EDTA LysC Acidic and 5a- EDTA+GuHCl Acidic were excluded from the study as no reliable MS spectra were generated.

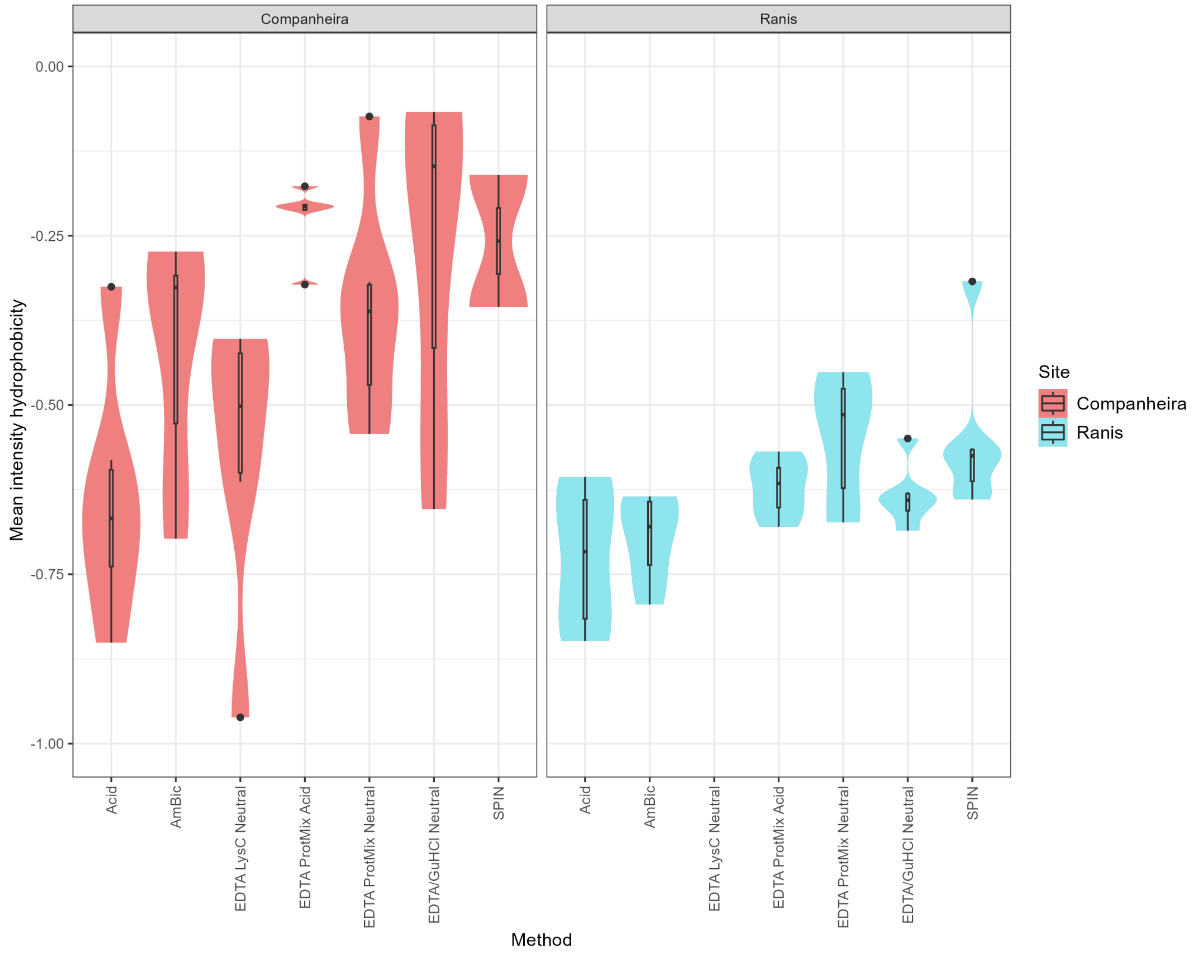

Supplementary Figure S9. Average peptide hydropathy (GRAVY) score weighted by peptide intensity in the "specific" MaxQuant search per excavation site and extraction method; 1-Acid, 2-AmBic, 3b-EDTA LysC Neutral, 4a-EDTA Protease Mix Acidic, 4b-EDTA Protease Mix Neutral, 5b-EDTA+GuHCl Neutral, 6-SPIN. GRAVY score is the sum of the hydropathy values for all the amino acids in a protein divided by the total number of residues in it. Negative GRAVY value indicates that the protein is non-polar (hydrophobic peptides) and positive value indicates that the protein is polar (hydrophilic peptides). EDTA LysC Neutral (3b) extraction method generated no results for Ranis specimens. Extraction methods 3a- EDTA LysC Acidic and 5a- EDTA+GuHCl Acidic were also excluded from the study as no reliable MS spectra were generated.

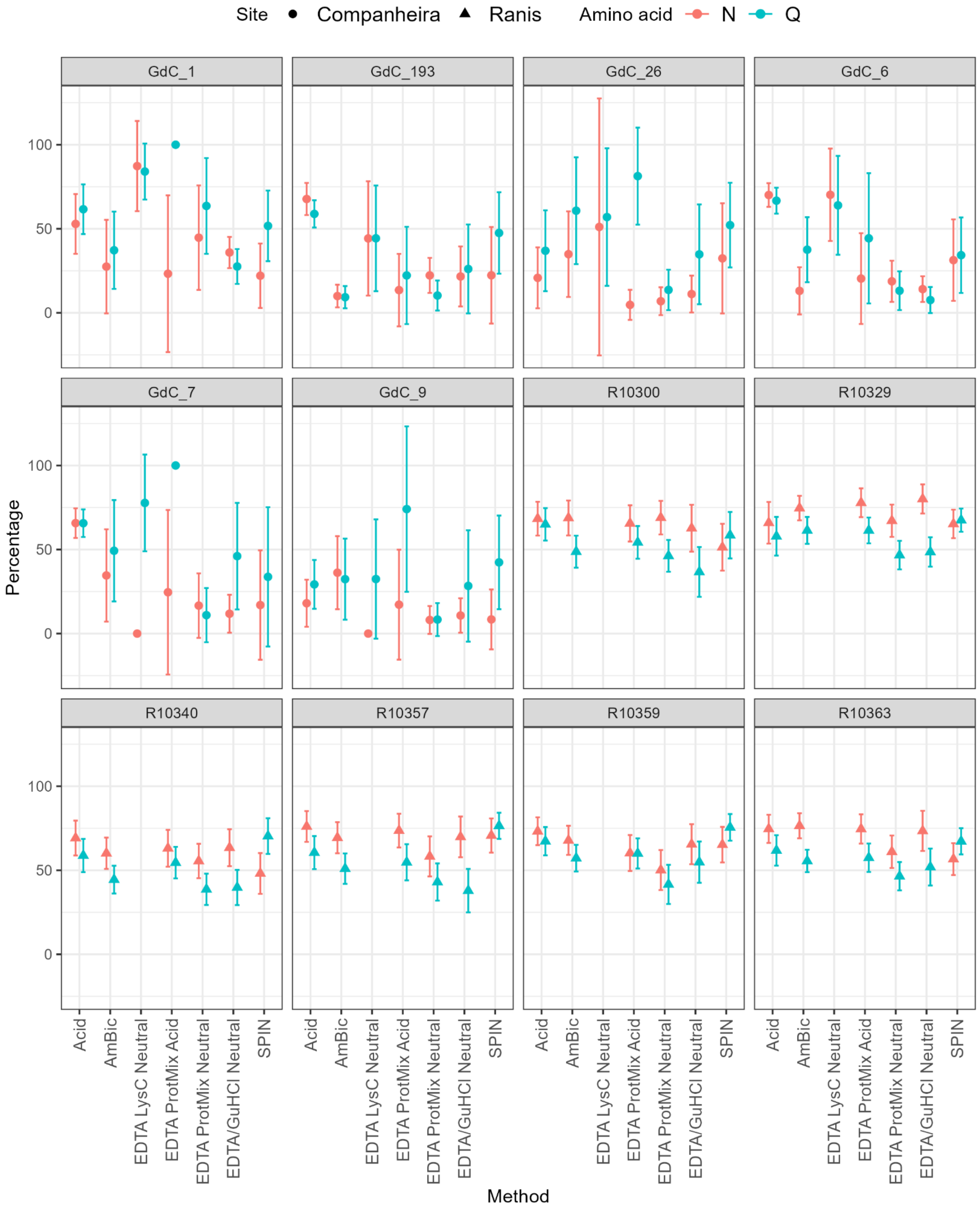

Supplementary Figure S10. Amino acid deamidation in the "semi-specific" MaxQuant search" for each sample by extraction method; 1-Acid, 2-AmBic, 3b-EDTA LysC Neutral, 4a-EDTA Protease Mix Acidic, 4b-EDTA Protease Mix Neutral, 5b-EDTA+GuHCl Neutral, 6-SPIN. The y-axis represents the percentage of deamidation rate for Q and N respectively for Ranis for each sample per extraction method, where 100% indicates complete deamidation and 0% indicates no deamidation. Error bars represent 2SD. EDTA LysC Neutral (3b) extraction method generated no results for Ranis specimens. Extraction methods 3a- EDTA LysC Acidic and 5a- EDTA+GuHCl Acidic were also excluded from the study as no reliable MS spectra were generated.

Abbreviations

ZooMS	Zooarchaeology by Mass spectrometry
SPIN	Species by proteome investigation
PTMs	Post-translational modifications
LP	Late Pleistocene
HPLC	High-performance liquid chromatography
MS	Mass spectrometry
MALDI-TOF MS	Matrix-assisted laser desorption/ionization- Time of flight Mass spectrometry
PMF	Peptide mass fingerprinting
DIA	Data-independent acquisition
DDA	Data-dependent acquisition
GdC	Gruta da Companheira
HTS	High-throughput shotgun proteomics
SAP	Single amino acid polymorphism
LC-MS/MS	Liquid-chromatography tandem mass spectrometry
m/z	Mass to charge
SWATH-MS	Sequential window acquisition of all theoretical fragment ion spectra
HDMSE	High-resolution MS^E
IT	Ion trap
NCE	Normalized collision energy
AGC	Automatic gain control
AIF	All-ion fragmentation
PAC	Protein aggregation capture
EDTA	Ethylenediaminetetraacetic acid
Try	Trypsin
LysC	rLys-C
NP-40	Nonyl phenoxypolyethoxylethanol
EthOH	Ethanol
UHQ	Ultra-high quality
TCEP	Tris(2-carboxyethyl) phosphine

NEM	N-ethylmaleimide
GuHCl	Guanidine hydrochloride
Tris-HCl	Tris-(hydroxymethyl)-aminomethane and hydrochloric acid
iPrOH	Isopropyl alcohol
AmBic	Ammonium bicarbonate
TFA	Trifluoroacetic acid
ACN	Acetonitrile
HCl	Hydrochloric acid
RT	Room temperature
Gln/Q	Glutamine
Glu/E	Glutamic acid
NCPs	Non-collagenous proteins
Cys/C	Cysteine
Lys/K	Lysine
Arg/R	Arginine
Asn/N	Asparagine
Met/M	Methionine
